# Association of Penicillin Allergy Label With Post‐COVID‐19 Condition and Subsequent Clinical Outcomes: A Population‐Based Cohort Study

**DOI:** 10.1111/cea.70297

**Published:** 2026-04-06

**Authors:** Ubonphan Chaichana, Kenneth K. C. Man, Chengsheng Ju, Yogini H. Jani, Li Wei

**Affiliations:** ^1^ Research Department of Practice and Policy UCL School of Pharmacy London UK; ^2^ Centre for Medicines Optimisation Research and Education University College London Hospitals National Health Service (NHS) Foundation Trust London UK; ^3^ Centre for Safe Medication Practice and Research, Department of Pharmacology and Pharmacy, li Ka Shing Faculty of Medicine The University of Hong Kong, Hong Kong Special Administrative Region China; ^4^ Institute of Cardiovascular Science, University College London London UK

**Keywords:** clinical outcomes, penicillin allergy, post‐COVID‐19 condition

## Abstract

- Penicillin allergy labelling was associated with increased risk of post‐COVID‐19 condition and subsequent clinical outcomes.
- The increased risk of subsequent clinical outcomes was not mediated by PCC.

To the Editor,

Penicillin allergy labelling is common [1] and has been linked to worse outcomes in acute COVID‐19 [2]. Its impact on post‐COVID‐19 condition (PCC) and subsequent clinical outcomes, including nonfatal stroke, myocardial infarction, and all‐cause mortality, remains unclear. We aimed to evaluate whether penicillin allergy labelling is associated with increased risk of PCC and subsequent clinical outcomes, and whether PCC mediates these associations.

We conducted a population‐based cohort study using UK primary care data from the Clinical Practice Research Datalink (CPRD) Aurum [3] (March 2020–July 2023). Adults (≥ 18 years) with confirmed SARS‐CoV‐2 infection and ≥ 1 year of GP registration were included. The exposure was the individuals with a presence of a penicillin allergy label before the index date (90 days after the Covid‐19 diagnosis date), identified through medical records using Read Codes (TJ0..00, TJ00000, TJ00z00) or the ICD‐10 code (Z88.0). The primary outcome was the occurrence of PCC defined using diagnostic codes or ≥ 1 WHO‐listed symptom occurring 90–365 days post‐infection, absent in the preceding 180 days. The secondary outcome was subsequent clinical outcomes ≥ 90 days post‐COVID. Eligible individuals were followed from the index date until they developed PCC symptoms, were lost to follow‐up, died, or reached the end of the study period (1 year Post‐COVID Diagnosis), whichever occurred first. In the mediation analysis, the follow‐up was started at 90 Days Post‐COVID Diagnosis until they developed subsequent clinical outcomes, died, or reach the end of study period (31 July 2023), whichever occurred first. The cumulative incidence of PCC and subsequent clinical outcomes was compared between individuals with and without a penicillin allergy label using Cox proportional hazards models. Hazard ratios (HRs) and risk differences with 95% confidence intervals (CIs) were estimated after adjustment using propensity score fine stratification weighting based on the average treatment effect [4]. Covariate balance was assessed using standardised mean differences (SMDs), with values < 0.2 indicating acceptable balance. Mediation analysis was conducted to evaluated whether PCC mediated the association between penicillin allergy labelling and subsequent clinical outcomes. This study was approved by the CPRD Research Data Governance Expert Review Committee (Protocol reference ID: 23_002694).

Among 1,630,812 individuals with recorded SARS‐CoV‐2 infection, 1,587,288 met eligibility criteria, including 36,350 with a penicillin allergy label. The mean age was 43.9 (SD 16.3) years. After propensity score fine stratification weighting, baseline characteristics were well balanced (all SMDs < 0.2).

At 1 year, individuals with a penicillin allergy label had a higher risk of PCC compared with those without a label (incidence rate 36.35 versus 26.48 per 100 person‐years; Figure 1A); risk difference 10.8%, 95% CI 10.6%–11.1%; adjusted HR 1.09, 95% CI 1.07–1.12. Findings were consistent across subgroups. Sensitivity analyses, including complete‐case analysis (aHR 1.09, 95% CI 1.06–1.12) and an alternative SNOMED‐coded PCC definition (aHR 1.13, 95% CI 1.04–1.23), yielded similar results.

**FIGURE 1:**
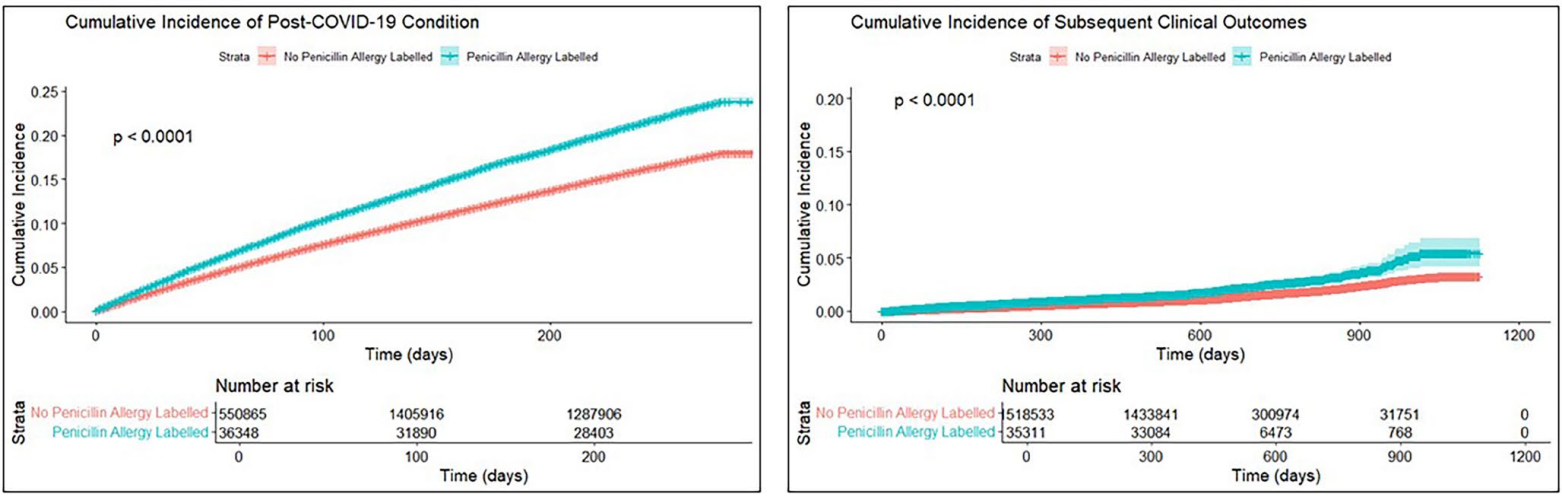
(A) The cumulative incidence curve of PCC events comparing individuals with and without a penicillin allergy label, per 100 person‐years. (B) The cumulative incidence curve of subsequent clinical outcomes comparing individuals with and without a penicillin allergy label, per 100 person‐years.

After excluding individuals with prior stroke or myocardial infarction, patients with a penicillin allergy label also had a higher risk of subsequent clinical outcomes occurring ≥ 90 days after COVID‐19 diagnosis (incidence rate 1.15 vs. 0.73 per 100 person‐years (Figure 1B); adjusted HR 1.10, 95% CI 1.01–1.21).

Mediation analysis showed a significant total effect of penicillin allergy label on adverse clinical outcomes (OR 1.13, 95% CI 1.02–1.26). However, the natural indirect effect via PCC was not significant, indicating that PCC did not meaningfully mediate this association.

To our knowledge, no previous studies have evaluated the association between penicillin allergy labelling and PCC or subsequent clinical outcomes. Prior research has focused on short‐term outcomes within 30 days and reported worse acute COVID‐19 outcomes among patients with a penicillin allergy label but no significant increase in 30‐day mortality [2]; notably, its point estimate was similar to ours. However, that study examined hospitalised patients and short‐term outcomes, whereas our cohort was largely primary care–based with a mean follow‐up of 1.37 years. Consistent with previous literature, penicillin allergy labelling has also been associated with increased cardiovascular disease and mortality risk [5, 6, 7].

Several mechanisms may explain our findings. Patients with a penicillin allergy label are more likely to receive alternative or broad‐spectrum antibiotics, which may increase the risk of antimicrobial resistance resulting in cardiovascular complications [8, 9]. Psychosocial factors may also contribute. We acknowledge that social determinants, health literacy, and health anxiety could mediate or confound the observed associations. Although direct measures of health literacy are not available in the CPRD database, we adjusted for available proxies related to healthcare‐seeking behaviour and psychosocial factors to mitigate potential confounding. Additionally, subgroup analysis stratified by anxiety versus non‐anxiety status yielded results consistent with the main analysis, supporting the robustness of our findings.

Strengths of this study include its large, nationally representative primary care cohort and evaluation of long‐term outcomes. Limitations include potential under ascertainment and misclassification of PCC, incomplete allergy documentation, possible residual misclassification of true penicillin allergy status, and limited statistical power in some subgroups. Despite these limitations, our findings suggest that penicillin allergy labelling may represent an independent risk marker for subsequent clinical outcomes following COVID‐19. Nevertheless, because many individuals with a recorded penicillin allergy label may not have a true allergy when formally evaluated, these results should be interpreted with caution.

Our study suggests that penicillin allergy labelling may increase the risks of PCC and subsequent clinical outcomes, and this association was not mediated by PCC, highlighting the importance of accurate allergy assessment and documentation.

## Author Contributions

L.W., K.K.C.M. and U.C. conceived the study. U.C. and C.J. carried out the statistical analysis and interpreted the data. U.C. wrote the first draft of the manuscript. All authors critically revised and edited the manuscript and approved the final version for submission.

## Funding

The authors have nothing to report.

## Conflicts of Interest

U.C. reported receiving a scholarship from the Royal Thai Government outside the submitted work. K.K.C.M. reports grants from the CW Maplethorpe Fellowship, the European Union Horizon 2020, the UK National Institute of Health Research, the South Korea Ministry of Food and Drug Safety, the Hong Kong Research Grant Council, and the Hong Kong Innovation and Technology Commission, consultancy from IQVIA, AstraZeneca, outside of the submitted work. L.W. reported receiving grants from the National Institute Health Research Health Technology Assessment, Diabetes UK, The Cure Parkinson's Trust outside the submitted work. No other disclosures were reported.

## Data Availability

This study is based on data from the CPRD, obtained under licence from the UK MHRA. The data are not publicly available and can only be accessed by approved researchers who have obtained the necessary permissions from CPRD. Access to CPRD data requires a protocol submission and approval by the Independent Scientific Advisory Committee (ISAC). The authors do not have permission to share the data. Repository materials can be found at https://doi.org/10.5281/zenodo.19005269.
